# Diagnostic Capability of OCTA-Derived Macular Biomarkers for Early to Moderate Primary Open Angle Glaucoma

**DOI:** 10.3390/jcm13144190

**Published:** 2024-07-18

**Authors:** Alice Verticchio Vercellin, Alon Harris, Francesco Oddone, Carmela Carnevale, Brent A. Siesky, Julia Arciero, Brendan Fry, George Eckert, Paul A. Sidoti, Gal Antman, Denise Alabi, Janet C. Coleman-Belin, Louis R. Pasquale

**Affiliations:** 1 Department of Ophthalmology, Icahn School of Medicine at Mount Sinai, New York, NY 10029, USA; alice.verticchio@mssm.edu (A.V.V.); brent.siesky@mssm.edu (B.A.S.); psidoti@nyee.edu (P.A.S.); antmangal@gmail.com (G.A.); denise.alabi@icahn.mssm.edu (D.A.); janet.coleman-belin@icahn.mssm.edu (J.C.C.-B.); louis.pasquale@mssm.edu (L.R.P.); 2 Glaucoma Unit, IRCCS—Fondazione Bietti, 00198 Rome, Italy; oddonef@gmail.com (F.O.); carmela.carnevale@fondazionebietti.it (C.C.); 3 Department of Mathematical Sciences, Indiana University Indianapolis, Indianapolis, IN 46202, USA; jarciero@iu.edu; 4 Department of Mathematics and Statistics, Metropolitan State University of Denver, Denver, CO 80204, USA; bfry2@msudenver.edu; 5 Department of Biostatistics and Health Data Science, Indiana University School of Medicine, Indianapolis, IN 46202, USA; geckert@iu.edu; 6 New York Eye and Ear Infirmary of Mount Sinai, New York, NY 10003, USA; 7 Department of Ophthalmology, Rabin Medical Center, Petach Tikwa 4941492, Israel; 8 Faculty of Medicine, Tel Aviv University, Tel Aviv 69978, Israel

**Keywords:** primary open-angle glaucoma, optical coherence tomography angiography, macula, superficial capillary plexus

## Abstract

**Background/Objectives**: To investigate macular vascular biomarkers for the detection of primary open-angle glaucoma (POAG). **Methods**: A total of 56 POAG patients and 94 non-glaucomatous controls underwent optical coherence tomography angiography (OCTA) assessment of macular vessel density (VD) in the superficial (SCP), and deep (DCP) capillary plexus, foveal avascular zone (FAZ) area, perimeter, VD, choriocapillaris and outer retina flow area. POAG patients were classified for severity based on the Glaucoma Staging System 2 of Brusini. ANCOVA comparisons adjusted for age, sex, race, hypertension, diabetes, and areas under the receiver operating characteristic curves (AUCs) for POAG/control differentiation were compared using the DeLong method. **Results:** Global, hemispheric, and quadrant SCP VD was significantly lower in POAG patients in the whole image, parafovea, and perifovea (*p* < 0.001). No significant differences were found between POAG and controls for DCP VD, FAZ parameters, and the retinal and choriocapillaris flow area (*p* > 0.05). SCP VD in the whole image and perifovea were significantly lower in POAG patients in stage 2 than stage 0 (*p* < 0.001). The AUCs of SCP VD in the whole image (0.86) and perifovea (0.84) were significantly higher than the AUCs of all DCP VD (*p* < 0.05), FAZ parameters (*p* < 0.001), and retinal (*p* < 0.001) and choriocapillaris flow areas (*p* < 0.05). Whole image SCP VD was similar to the AUC of the global retinal nerve fiber layer (RNFL) (AUC = 0.89, *p* = 0.53) and ganglion cell complex (GCC) thickness (AUC = 0.83, *p* = 0.42). **Conclusions**: SCP VD is lower with increasing functional damage in POAG patients. The AUC for SCP VD was similar to RNFL and GCC using clinical diagnosis as the reference standard.

## 1. Introduction

Peripapillary retinal nerve fiber layer (RNFL) thickness and optic nerve head (ONH) biomarkers are hallmark structural indicators for glaucomatous optic nerve damage [1]. Optical coherence tomography (OCT) angiography (OCTA) allows for the visualization of attendant ONH, peripapillary, and macular vascular tissues [2,3]. While strong evidence supports the diagnostic accuracy of assessing the ONH and RNFL structure with OCT, loss of capillaries and vascular density (VD) in ONH and peripapillary tissues are also strongly associated with POAG [2,3,4]. Researchers using OCTA have specifically linked rapid initial ONH VD loss with faster visual field progression rates [5]. ONH VD has been previously demonstrated to have a similar diagnostic accuracy to RNFL thickness for diagnosing glaucoma [6,7]. Pilot artificial intelligence (AI) and machine learning (ML) neural network models have indicated that the inclusion of ONH VD biomarkers may enhance POAG diagnosis compared to only using RNFL and ONH structural features [8,9,10,11].

Although significantly fewer data are available than for peripapillary tissues, the inclusion of macular structure and VD biomarkers may also provide insights into early POAG. Structural parameters at the level of the macula, including the ganglion cell complex (GCC) thickness (thickness of the three innermost macular layers, the retinal nerve fiber layer (RNFL), the ganglion cell layer and inner plexiform layers), and VD represent novel biomarkers of interest. Reductions in macular thickness have been previously linked with lower OCTA-measured VD in regions of the retina and ONH of POAG patients [3,12,13,14,15]. Rapid GCC thinning has also been specifically associated with faster rates of central visual field decline in glaucoma patients [16]. The importance of macular parameters as biomarkers of disease have been suggested in POAG patients with particular ocular characteristics, with data suggesting for example that the diagnostic accuracy of macular RNFL thickness is higher in high myopes than in non-high myopia patients [17,18]. Pilot work has correlated a loss of macular VD with the extent and severity of visual field damage [13,14,15,19,20]. The association between visual field damage and loss of OCTA-assessed macular VD could be the expression of dysfunctional retinal ganglion cells (not yet atrophied) [13]. Notably, macular VD loss and GCC thinning have been shown to be detectable in preperimetric and early-stage POAG [21,22].

Together, these data suggest the utilization of OCTA-assessed macular VD biomarkers may improve diagnostic accuracy and help facilitate earlier, more individualized management options for POAG. Most studies investigating macular VD parameters and functional damage, however, have confined the analysis to the superficial capillary plexus (SCP) [14,22,23,24] and/or advanced [20,25,26] glaucoma. Current data are limited on the diagnostic accuracy of macular deep capillary plexus (DCP) VD deficits [27,28] in early-stage POAG [22]. In addition, most studies to date have focused on average VD values at the level of the global macular region and have not evaluated specific regional deficits at the level of the parafoveal or perifoveal regions or the hemispheric and/or quadrant level. In clinical practice, often only the global (average) values of structural and vascular biomarkers are analyzed and used to make therapeutic decisions. However, global values within normal limits may hide regional hemodynamic or structural damage that may lead to disease progression in glaucoma eyes. This stresses the importance of novel approaches that assess not only global (average) values of OCTA-derived macular biomarkers but also their regional levels (in terms of hemispheres/quadrants and parafovea/perifovea). Newer OCTA applications are also allowing for the quantification of structural and hemodynamic parameters related to the foveal avascular zone (FAZ) including the area, perimeter, VD, and flow within deeper layers (outer retina and choriocapillaris) [29,30,31,32]. In a recent study using a generalized linear mixed-effect model to differentiate FAZ progressors versus non-FAZ progressors (defined as slope >95 percentile of healthy tested), faster GCC thinning and faster visual field loss were found in eyes with FAZ progressors compared to non-FAZ progressors [33]. Together, these pilot data suggest that the potential diagnostic abilities of macular VD parameters for detecting early-stage POAG are compelling, yet the link between macular VD biomarkers and visual field damage remains insufficiently described.

This pilot study investigates differences in OCTA-derived macular VD biomarkers in early to moderate POAG patients and non-glaucomatous controls and assesses their relationship with visual field damage. This study is among the first to quantify macular VD at the level of the SCP and DCP at a global, hemispheric, and quadrant level and to evaluate differences between POAG patients and controls in structural and hemodynamic parameters in the FAZ as well as in the outer retinal layer and the choriocapillaris.

## 2. Materials and Methods

In this pilot cross-sectional study 56 POAG patients and 94 controls were enrolled between March 2021 and September 2023 at the Icahn School of Medicine at Mount Sinai (Department of Ophthalmology), New York, NY, United States. A written informed consent was signed by all study subjects before the start of this study. The study was conducted in accordance with the Declaration of Helsinki, and the study protocol was approved by the Institutional Review Board of Icahn School of Medicine at Mount Sinai, New York, NY, USA (protocol code: Study-20-00198; date of approval: 19 April 2020).

The eligibility criteria and the details of the two-hour study visit are described in detail in [12]. In summary, one randomly selected (coin flip) qualified eye was included in the study for each participant. Participants had to be 21 years old or older. A fellowship-trained glaucoma specialist confirmed POAG in the eye to be studied based on the presence of an open angle and characteristic structural and functional glaucomatous optic disc damage with reliable visual fields (<33% fixation losses, <20% false-positive, <20% false-negative) and strong OCTA signal strength. The control group included subjects with normal ONH and RNFL structure, visual fields free from defects, and eyes free from all ocular disease including POAG. The criterial of exclusion for study groups [12] were refractive error greater or less than 9 diopters in spherical equivalent; evidence of exfoliation or pigment dispersion; eye disease other than glaucoma; use of ophthalmic medications (other than for lowering of intraocular pressure (IOP), neurological disease, unreliable eye exams; and uncontrolled cardiovascular, renal, or pulmonary disease.

Humphrey field analyzer II using the 24-2 Swedish interactive threshold algorithm standard (white III stimulus) V.4.1 (Carl Zeiss Mediatec, Dublin, CA, USA) was used to assess visual function. The MD, PSD, and visual field index (VFI) were recorded. POAG patients were categorized in functional disease severity based on Glaucoma Staging System 2 (GSS2) of Brusini [34] as stage 0 (S0, no defect or minimal), borderline, stage 1 (S1, early defect), stage 2 (S2, moderate defect), stage 3 (S3, advanced), and stage 4 (S4, very advanced). As our study primarily targeted early POAG, none of the study participants were in stage 5.

**Figure 1:**
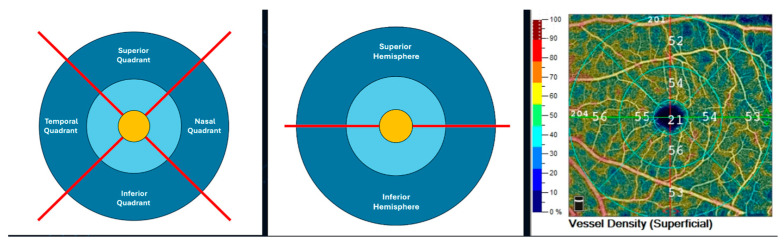
**Left** and **center:** Schematic representation of the 6 mm ETDRS grid derived from the 6 mm Angio Retina scan (right eye). The ETDRS grid is comprised of 3 concentric rings: 1 mm center (macula center, orange), 1–3 mm (parafovea, light blue), and outer ring of 3–6 mm diameters (perifovea, dark blue). The parafovea and perifovea rings are divided into 4 quadrants (temporal, superior, nasal, and inferior; **left**) and 2 hemispheres (superior and inferior) divided by a horizontal line through the foveal center (**center**). **Right**: Color-coded superficial capillary plexus vessel density map (right eye).

OCTA imaging (RTVue XR Avanti System Version 2018.1.1.63, Optovue Inc., Fremont, CA, USA) with an AngioAnalytics^TM^ licensed upgrade was assessed in all study eyes. Separate VD analysis computed as a percentage of the area occupied by OCTA-detected vasculature at the level of the macula was automatically provided [29]. Macular VD was assessed via the 6.0 mm HD AngioRetina scan in the SCP in the superficial slab (with the upper limit the inner limiting membrane and lower limit the inner plexiform layer −10 μm) and in the DCP in the deep slab (with upper limit the inner plexiform layer −10 μm and lower limit the outer plexiform layer +10 μm). VD was assessed in the ETDRS grid including 3 concentric rings [29]: macular center (1 mm diameter, depicted in orange color in Figure 1), parafovea (1–3 mm diameters, depicted in light blue color in Figure 1), and perifovea (3–6 mm diameters, depicted in dark blue color in Figure 1).

OCTA-derived biomarkers were also assessed in the FAZ, which represents the capillary-free center of the macula. The following FAZ measurements were assessed in the “retina slab” (inner limiting membrane to outer plexiform layer +10 μm): FAZ area (mm^2^), FAZ perimeter (mm), and FD (VD in % of the 300 μm width ring surrounding the FAZ calculated by dividing the number of vessels pixels by the total number of pixels, multiplied by 100%). We also assessed the flow area in the outer retina slab (from outer plexiform layer +10 μm to Bruch membrane −10 μm) and in the choriocapillaris (in the choriocapillaris slab, from the Bruch membrane −10 μm to Bruch membrane +30 μm were also assessed in mm^2^ (predefined circle area: 8.045 mm^2^)). In addition to the macular OCTA parameters, the average (global) RNFL thickness was assessed via the 4.5 mm HD Angio Disc scan in the peripapillary region (defined by two rings of 2 mm and 4 mm centered on the disc center) [29]. The macular GCC thickness was measured via the GCC scan. Images were included in the analysis only if the quality was optimal (signal strength index for the GCC scan > 50; scan quality indicator for the OCTA scans > 6).

ANCOVA comparisons adjusted for age, sex, race, hypertension, and diabetes were used to test for differences between subjects, and areas under the receiver operating characteristic curves (AUROCs) were calculated. AUROC comparisons were made using the DeLong method. *p* < 0.05 was considered statistically significant. All *p*-values are presented without adjustment for multiple testing. These analyses were performed in a relatively small study to explore the utility of macular OCTA parameters, and significant results will need to be verified in additional and/or larger studies.

## 3. Results

Table 1 shows the demographic, ocular, and systemic parameters for POAG patients and controls. POAG patients were significantly older than control subjects (*p* < 0.001) and significantly more had hypertension (*p* = 0.028). POAG patients had significantly worse VF MD, PSD, and VFI compared to control subjects (*p* < 0.05, Table 1). No significant differences between groups were found for sex ratio, body mass index, IOP, systolic and diastolic blood pressure, mean arterial pressure, ocular perfusion pressure, systolic and diastolic ocular perfusion pressure, or heart rate (all *p* > 0.05, as described in Table 1). The comparisons between outcomes were adjusted for age, sex, race, hypertension, and diabetes.

Table 2 shows the difference in OCTA-derived biomarkers between POAG patients and controls. RNFL and GCC thickness were significantly lower in POAG patients compared to control subjects (*p* < 0.001). Mean SCP VD was significantly lower in POAG patients compared to controls in the whole image, parafovea, and perifovea at a global, hemispheric, and quadrant level (all *p* < 0.001). No statistically significant differences were found between POAG patients and controls for DCP VD, FAZ parameters, and retinal and choriocapillaris flow area (*p* > 0.05).

**Figure 2:**
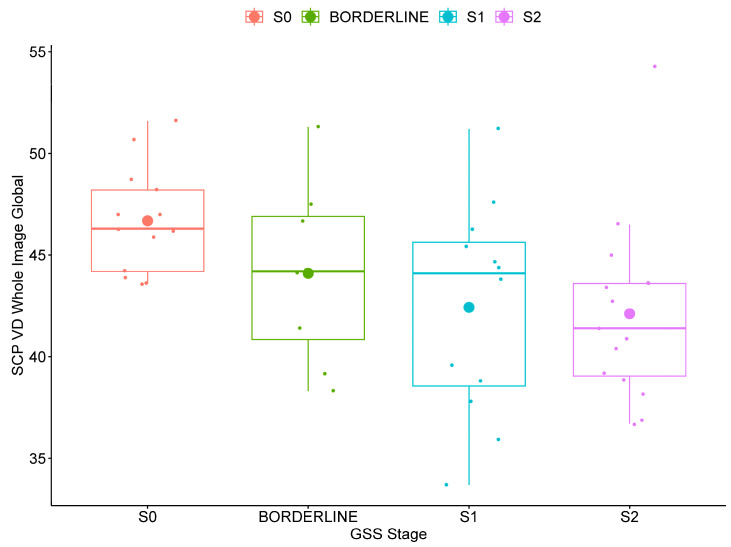
Box plot of whole image global superficial capillary plexus vessel density (SCP VD) in open-angle glaucoma patients stratified for functional severity based on the Glaucoma Staging System 2 (GSS2) of Brusini in stage 0 (S0, no defect or minimal), borderline, stage 1 (S1, early defect), and stage 2 (moderate defect).

**Figure 3:**
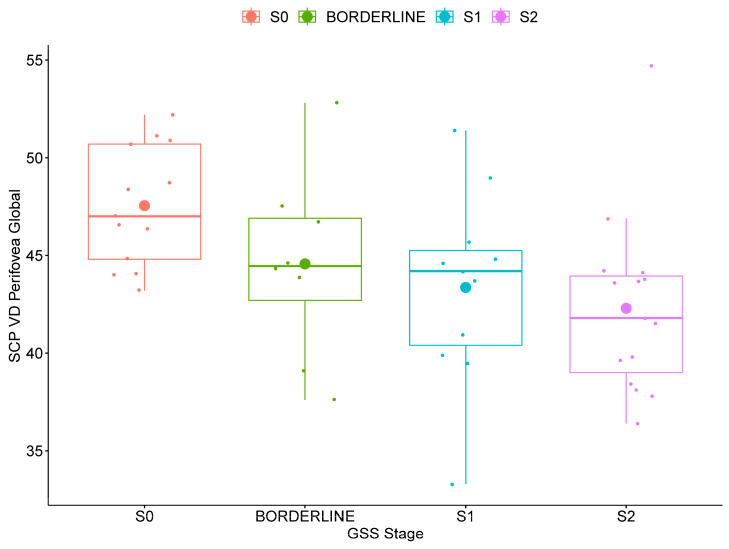
Box plot of perifoveal global superficial capillary plexus vessel density (SCP VD) in primary open-angle glaucoma patients stratified for functional severity based on the Glaucoma Staging System 2 (GSS2) of Brusini in stage 0 (S0, no defect or minimal), borderline, stage 1 (S1, early defect), and stage 2 (moderate defect).

SCP VD was significantly lower in POAG patients with increased functional disease severity (GSS2 stage 2 compared to stage 0) for the whole image (global, superior and inferior hemispheres) and several (global, superior and inferior hemispheres, superior, inferior and temporal quadrants) perifoveal regions (*p* ≤ 0.038). The box plots of the global SCP VD in POAG patients stratified for functional disease severity are shown in Figure 2 for the whole image and in Figure 3 for the perifoveal region.

**Figure 4:**
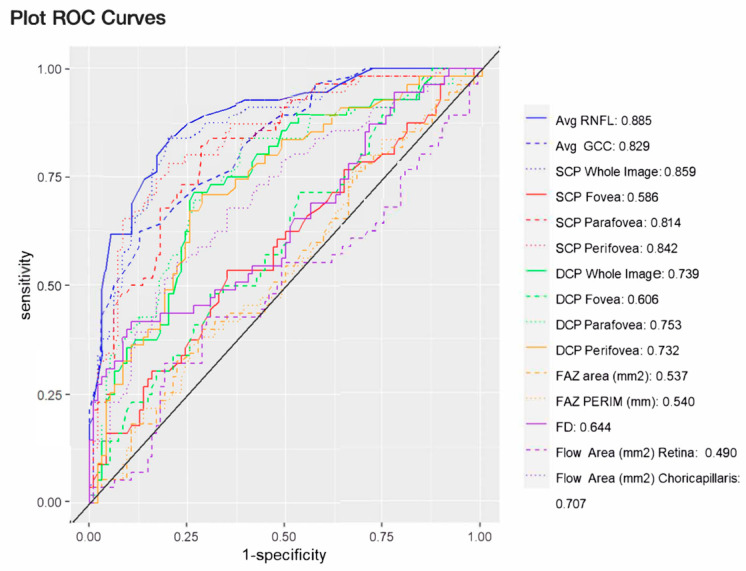
Areas under the receiver operating characteristic curves of optical coherence angiography derived structural and hemodynamic parameters. Avg: average; DCP: macular deep capillary plexus vessel density; FAZ: foveal avascular zone; FAZ PERIM: FAZ perimeter; FD: FAZ vessel density; GCC: ganglion cell complex thickness; RNFL: retinal nerve fiber layer thickness; SCP: macular superficial capillary plexus vessel density; SD: standard deviation; VD: vessel density.

The AUC of the SCP VD whole image (0.86) was significantly higher than the AUC of the DCP VD (*p* ≤ 0.018), FAZ parameters (*p* < 0.001), and retinal (*p* = 0.006) and choriocapillaris flow area (*p* < 0.001), as depicted in Figure 4. The best-performing macular OCTA biomarker (whole image SCP VD) was similar to the AUC of global RNFL (AUC = 0.89, *p* = 0.53) and GCC thickness (AUC = 0.83, *p* = 0.42).

## 4. Discussion

Traditional metrics for POAG consider changes in the ONH structure, attrition of the RNFL, and visual field loss. Advancements in ocular imaging including novel applications of OCTA are facilitating the quantification of vascular biomarkers alongside structural outcomes within various anatomical regions. Although limited in scope, structural and hemodynamic changes in the macula have been linked to POAG disease. OCTA-assessed macular VD biomarkers have demonstrated low variability over time, and changes in macular VD of ~5% (and GCC > 2 µm) have both been linked to disease progression [35]. Macular VD and GCC have been shown to be complementary biomarkers for detecting central visual field defects in eyes with advanced glaucoma [25]. Ghahari et al. found ONH and macula OCTA VD to both be specifically associated with the severity of functional loss [26]. Few studies, however, have investigated both the superficial and deep capillary macular plexus as well as the FAZ parameters and vascular biomarkers of the choriocapillaris and outer retina in relation to field damage in early-stage POAG patients [24,27,28,31,32]. In this analysis, we investigated the differences in OCTA-derived VD in the SCP and DCP, FAZ parameters, and flow area in the outer retina and choriocapillaris in early-stage POAG patients categorized based on functional disease severity and control subjects. This approach allowed us to quantify and describe specific connections of macular VD and visual field defects in the early disease stages of POAG.

In our analysis, POAG patients had significantly reduced global RNFL and GCC thickness and SCP VD in the whole image as well as at the hemispheric and quadrant levels compared to controls (all *p* < 0.001, Table 1). VD in the DCP, while reduced in early-stage POAG patients, was not significantly different when compared to control subjects (Table 1, *p* > 0.05). These data agree with previously published data in the literature showing reduced SCP VD in POAG patients compared to controls assessed by OCTA [14,20,23]. Our results also agree with data suggesting more SCP VD loss than DCP loss in early POAG. Previously, Wu et al. found that lower baseline superficial but not deep parafoveal VD was associated with faster parafoveal GCC thinning in POAG patients [28]. These results are also consistent with the fact that the SCP supplies the RNFL and GCC, which are documented areas of glaucomatous damage [27]. As SCP VD supplies the anatomic structures affected by glaucoma, it may represent an important biomarker of disease. Notably, our patients had early-stage disease without significant visual field loss; thus, these results suggest SCP VD changes may occur before DCP loss.

In POAG patients, SCP VD was also significantly lower with increased functional disease severity for the whole image (Figure 2) and for several perifoveal regions (Figure 3), while no differences were found in the parafoveal regions. Previous studies conducted in preperimetric and early glaucoma have similarly found that SCP VD loss occurred predominantly in the perifoveal region with less microvascular damage at the level of the parafovea [12,22,36]. Yarmohammadi et al. [13] compared perifoveal SCP VD hemifield differences in 58 POAG patients with visual field defects confined to one hemifield. The researchers specifically found that perifoveal SCP VD loss in each hemiretina was associated with the extent of perimetric damage in the corresponding hemifield. Previous pilot work suggests the potential importance of macular outcomes in the early stages of POAG disease [21]. In agreement with previous studies [12,22,36], our findings suggest that a loss of SCP VD may occur first in the peripheral (perifovea) region of the macula in patients with early disease.

To quantify the impact between early and moderate POAG disease stages, we used the Glaucoma Staging System 2 of Brusini [34] based on both MD and PSD to stratify the POAG patients for functional disease severity. This approach allowed us to identify that SCP VD was significantly lower in POAG patients with moderate defects (GSS2 stage 2) compared to patients with no or minimal defects (stage 0), while no significant differences were found in the DCP. Macular SCP VD loss has been previously shown to correlate with the extent and severity of visual field damage in glaucoma patients using different methods to classify functional disease severity [13,15,19,20]. In one example, Wu et al. found that SCP VD loss in 116 POAG eyes corresponded to disease severity based on the MD as the primary measure [15]. In our analysis, POAG patients in the S3 (advanced) and S4 (very advanced) stages were excluded due to their small sample size and our desire to focus on early-stage disease metrics. Previous work by Shin et al., utilizing the MD as functional damage criteria, found a strong utility of macular VD to monitor central VF sensitivity in moderate to advanced glaucoma [19]. However, the study was limited in that only the SCP was investigated. Larger, well-controlled studies are needed to further describe SCP and DCP VD loss in conjunction with visual field loss across the various stages of POAG.

In this analysis, we found no significant differences between POAG patients and controls for FAZ parameters area, perimeter, and FD (*p* > 0.05, Table 2). Similar results were previously reported by Oba et al., who did not find significant differences in the FAZ area when comparing POAG patients with controls (both preperimetric and early stage) [24]. These results, however, conflict with data from Zivkovic et al. who investigated FAZ parameters in 21 normal tension glaucoma (NTG) and 30 control eyes [31]. The authors found that NTG eyes had significantly larger FAZ mean vertical, horizontal, and maximum diameters and area as well as significantly reduced VD compared to the control group (all *p* < 0.001). The differences in the study results may be due to different inclusion criteria (i.e., NTG versus POAG) and the different OCTA methodologies and acquisition protocols utilized. For instance, in both our study and in the study by Oba et al. [24], Optovue OCTA was used with an acquisition of 6.0 mm HD AngioRetina scan to assess FAZ parameters, while Zivkovic et al. utilized ZEISS AngioPlex 3 × 3 macula scans [31]. These and other data [37] highlight that caution should be used when comparing data from different OCTA devices and point to the need for data harmonization when performing comparative analysis.

In our study, we demonstrated that the diagnostic accuracy of SCP VD (AUC = 0.859) in the whole image was significantly higher than DCP VD (AUC = 0.739, *p* < 0.001). These results agree with previous data from El-Nimri et al., who found that the AUC of the SCP whole image was significantly higher than that of the DCP for classifying glaucoma (SCP = 0.80 and DCP = 0.44, *p* < 0.001) [27]. We also found that the diagnostic accuracy of SCP VD in the whole image was similar to the AUC of global RNFL (*p* = 0.89) and GCC thickness (*p* = 0.83). Our results agree with previous studies that show GCC thickness and macula VD had similar diagnostic accuracy to discriminate preperimetric early glaucoma (all *p* > 0.05) [22]. In our analysis, we also found that the AUC of SCP VD in the whole image was significantly higher than the AUC of the FAZ parameters (*p* < 0.001, Figure 4). Previously, an enlarged FAZ area was associated with a greater risk of RNFL and macular-region ganglion cell-inner plexiform layer thinning but not with functional damage in patients with POAG and ocular hypertension [38]. In agreement, Kwon et al. also found that the AUC of the FAZ perimeter was similar to the one of the RNFL and macular ganglion cell-inner plexiform layer thickness for differentiating eyes with central visual field defects (all *p* > 0.05) [30]. The superior diagnostic ability of the FAZ perimeter shown in this analysis may be related to the fact that the other authors investigated eyes with central visual field defects, while our patients’ locations of VF damage varied. In addition, our OAG patients were early stage and did not include mixed cohorts or ocular hypertensives. Previous studies have identified how differences among patient criteria, including the race of subjects, may significantly affect macular OCTA VD biomarkers [39,40].

In contrast to inner layers, in this analysis, we found no significant differences in terms of the choriocapillaris and outer retinal layers between POAG patients and non-glaucomatous controls (*p* > 0.05, Table 1). The AUC of these biomarkers was significantly lower than the whole image SCP VD, RNFL, and GCC thickness (all *p* < 0.05). Our results agree with previous data from Milani et al., who did not find significant differences in the choriocapillaris perfusion area between eyes with glaucoma, ocular hypertension, and controls [32]. These limited data suggest that the macular choriocapillaris flow perfusion area is not as affected in early-stage POAG compared to the inner retinal and ONH tissues.

This study has several limitations to acknowledge. First, our sample was relatively small; future studies with an increased number of study participants may possibly allow for some of the comparisons to reach statistical significance. POAG patients were significantly older than the non-glaucomatous control subjects (Table 1), and aging has been associated with a reduction in macular VD [41]. To limit this impact, our results were statistically adjusted for age. The presence of diabetes mellitus and systemic hypertension may affect the OCTA-macular parameters assessed in this study [42,43]. For this reason, we adjusted statistically for systemic hypertension and diabetes mellitus to limit the effect of these confounding factors and excluded any participant with ocular complications (hypertensive retinopathy/diabetic retinopathy) that may have affected our results. For the same reason, we excluded patients with high myopia with myopic retinopathy or related retinal anatomic changes, as OCT/OCTA biomarkers may be affected by myopia [17,18]. Our study is also cross-sectional; thus, the relationship between SCP and DCP and other FAZ parameters and disease progression could not be evaluated. Only larger, well-controlled, prospective studies may reveal if macular OCTA VD biomarkers may be used as predictors for disease progression. Our study did not control for race and focuses on early-stage POAG, and the results with early to moderate functional damage may be of limited application to other advanced or very advanced POAG patients. Interestingly, macular OCTA parameters have been recently associated with visual acuity in moderate to advanced but not early glaucoma [44]. In our study, we used the GSS2 to classify the patients based on functional disease severity [34]. The use of different types of classifications systems, like the Hodapp–Parrish–Anderson classification, or the Global Staging System of Brusini among others [45,46,47], may have result in different findings. One strength of our study is that all OCT and OCTA exams were made by the same operator (A.V.V.) during the same session with limited time disruption between measurements. It is important to highlight that the ROC curve of the conventional OCT-derived global RNFL thickness was slightly higher (0.885) than the highest OCTA-assessed macular biomarker, which was found in the SCP whole image (0.859). This finding further confirms the well-established importance of using OCT-assessed RNFL thickness for monitoring of POAG disease.

## 5. Conclusions

In this analysis, we found that SCP VD was significantly lower in POAG patients compared to non-glaucomatous controls. In addition, there was a correlation between SCP VD and increasing functional severity based on visual field data. SCP VD had the highest performance among all OCTA-assessed macular vascular biomarkers and was statistically similar to RNFL and GCC thickness for diagnosing POAG. Most of the available literature is in agreement with our results, showing strong macular SCP VD utility with limited evidence of early DCP VD involvement. Due to similarities, small variations in study results are likely due to significant differences in study methodologies, patient populations, and macular scans rather than conflicting outcomes. Together, these results support other recent work suggesting that both structural and VD changes occur in the macular regions of early-stage POAG patients. SCP VD therefore represents a vascular biomarker that could be used to detect glaucoma in the early stages of the disease in combination or as an alternative to RNFL and GCC thickness. Non-invasively assessed macular VD biomarkers applied with highly specific analysis methodologies including AI and ML [8,9,10,11] may better inform clinicians and improve POAG diagnosis, especially for early-stage glaucoma patients.

## Figures and Tables

**Table 1:** Mean and standard deviation of demographic, ocular, and systemic parameters in control and primary open-angle glaucoma (POAG) patients. Comparisons between outcomes were adjusted for age, sex, race, hypertension, and diabetes. BMI: body mass index; bpm: beats per minute; DBP: diastolic blood pressure; DOPP: diastolic ocular perfusion pressure; HR: heart rate; IOP: intraocular pressure; MAP: mean arterial pressure; MD: mean deviation; OPP: ocular perfusion pressure; PSD: pattern standard deviation; SBP: systolic blood pressure; SD: standard deviation; SOPP: systolic ocular perfusion pressure; VF: visual field; VFI: visual field index; GSS2 stage: Glaucoma Staging System 2 (GSS2) of Brusini. *p* < 0.05 was considered statistically significant.

	Control	POAG	*p*-Value Control vs. POAG
Age at visit, years	40.3 (16.7)	63.7 (13.2)	*p* < 0.001
Sex, Male:Female	35%:65%	46%:54%	*p* = 0.17
Race	European descent: 44% African descent: 12% Latin descent: 20% Asian descent: 20% Other: 4%	European descent: 47% African descent: 21% Latin descent: 16% Asian descent: 16%	*p* = 0.50
BMI, kg/m^2^	26.4 (6.5)	25.1 (4.6)	*p* = 0.50
Diabetes	6%	16%	*p* = 0.056
Hypertension	18%	34%	*p* = 0.028
IOP, mmHg	14.7 (3.0)	16.0 (3.8)	*p* = 0.78
SBP, mmHg	120 (17)	123 (13)	*p* = 0.077
DBP, mmHg	75 (10)	76 (9)	*p* = 0.66
MAP, mmHg	90 (12)	92 (10)	*p* = 0.28
OPP, mmHg	45 (8)	45 (7)	*p* = 0.36
SOPP, mmHg	105 (17)	107 (13)	*p* = 0.086
DOPP, mmHg	61 (11)	60 (10)	*p* = 0.70
MOPP, mmHg	75 (12)	76 (10)	*p* = 0.32
Heart Rate, bpm	71 (12)	71 (13)	*p* = 0.75
Visual Field MD, decibel	−1.32 (1.83)	−2.72 (4.29)	*p* = 0.026
Visual Field PSD, decibel	2.14 (1.65)	3.40 (2.54)	*p* < 0.001
GSS2 Stage	S0: 13 Borderline: 8 S1: 12 S2: 15 S3: 2 S4: 1 Undetermined: 5		

**Table 2:** Mean and standard deviation of the optical coherence tomography parameters and comparisons (*p*-values) in control and open-angle glaucoma (POAG) patients. Comparisons between outcomes were adjusted for age, sex, race, hypertension, and diabetes. DCP: macular deep capillary plexus; FAZ: foveal avascular zone; FD: FAZ vessel density; GCC: ganglion cell complex; RNFL: retinal nerve fiber layer; SCP: macular superficial capillary plexus; SD: standard deviation; VD: vessel density. *p* < 0.05 was considered statistically significant.

		Control		POAG		*p*-Value Control vs. POAG
		Mean	SD	Mean	SD	
SCP VD (%)	Whole image	49.54	3.89	43.02	5.16	*p* < 0.001
	Whole image superior hemisphere	49.61	3.75	43.68	5.06	*p* = 0.003
	Whole image inferior hemisphere	49.47	4.19	42.33	5.50	*p* < 0.001
	Fovea global	20.15	7.35	17.65	7.92	*p* = 0.79
	Parafovea global	52.20	4.35	46.02	6.28	*p* = 0.011
	Parafovea superior hemisphere	52.30	4.17	46.46	6.19	*p* = 0.015
	Parafovea inferior hemisphere	52.09	4.87	45.57	6.84	*p* = 0.014
	Parafovea temporal quadrant	52.04	4.09	45.89	6.37	*p* = 0.003
	Parafovea superior quadrant	52.75	4.57	46.97	7.17	*p* = 0.05
	Parafovea nasal quadrant	51.72	4.51	45.56	6.77	*p* = 0.017
	Parafovea inferior quadrant	52.29	5.50	45.63	7.54	*p* = 0.039
	Perifovea global	50.06	4.17	43.62	5.28	*p* = 0.003
	Perifovea superior hemisphere	50.18	4.04	44.21	5.34	*p* = 0.007
	Perifovea inferior hemisphere	49.96	4.50	42.90	5.59	*p* = 0.001
	Perifovea temporal quadrant	45.85	4.75	39.55	5.30	*p* = 0.009
	Perifovea superior quadrant	50.42	4.48	43.97	5.85	*p* = 0.004
	Perifovea nasal quadrant	54.10	3.70	48.64	5.48	*p* = 0.022
	Perifovea inferior quadrant	49.94	4.83	42.47	5.90	*p* = 0.002
DCP VD (%)	Whole image	52.26	6.57	46.39	6.75	*p* = 0.18
	Whole image superior hemisphere	52.51	6.39	46.57	6.92	*p* = 0.12
	Whole image inferior hemisphere	52.01	6.98	46.18	6.85	*p* = 0.26
	Fovea global	37.91	8.63	34.43	8.80	*p* = 0.53
	Parafovea global	56.92	4.86	52.02	5.59	*p* = 0.07
	Parafovea superior hemisphere	57.21	4.67	52.36	5.82	*p* = 0.045
	Parafovea inferior hemisphere	56.62	5.34	51.85	5.68	*p* = 0.16
	Parafovea temporal quadrant	57.65	4.68	53.20	5.35	*p* = 0.049
	Parafovea superior quadrant	56.31	5.02	50.80	7.17	*p* = 0.05
	Parafovea nasal quadrant	58.18	4.63	53.99	5.65	*p* = 0.23
	Parafovea inferior quadrant	55.54	6.29	50.08	6.83	*p* = 0.15
	Perifovea global	53.52	7.05	47.50	7.43	*p* = 0.19
	Perifovea superior hemisphere	53.90	6.79	47.85	7.67	*p* = 0.16
	Perifovea inferior hemisphere	53.07	7.56	46.98	7.56	*p* = 0.26
	Perifovea temporal quadrant	55.32	6.50	49.34	6.69	*p* = 0.08
	Perifovea superior quadrant	53.36	7.62	47.12	8.20	*p* = 0.26
	Perifovea nasal quadrant	52.49	7.13	46.96	8.42	*p* = 0.22
	Perifovea inferior quadrant	52.79	8.42	46.52	8.04	*p* = 0.43
FAZ	FAZ area (mm^2^)	0.27	0.12	0.29	0.12	*p* = 0.19
	FAZ perimeter (mm)	1.99	0.45	2.06	0.46	*p* = 0.22
	FD (%)	54.37	4.55	50.57	6.40	*p* = 0.12
Flow Area (mm^2^)	Retina	0.70	0.35	0.74	0.40	*p* = 0.71
	Choriocapillaris	2.17	0.13	2.07	0.16	*p* = 0.60
RNFL thickness	Average RNFL thickness	100.33	9.02	82.33	12.48	*p* < 0.001
GCC thickness	Average GCC thickness	95.20	7.46	83.32	10.35	*p* < 0.001

## Data Availability

Data presented in the study are included in the article; further inquiries can be directed to the corresponding author.
